# 

**Published:** 1875-03-01

**Authors:** 


					﻿No. V.
MARCH 1, 1876.
Vol. XVI.
TZBZZE
Midical Iiaiiiss-
A
Stmi-fjoniHg Ifouijnal of Medical Sciences.
EDITED BY
N. S. DAVIS, M. D., and F. H. DAVIS, M. D.
Subscription Price, 1 hree Dollars per Annum, in advance.
Single Numbers, Fifteen Cents.
■ CHICAGO.
PUBLISHED AT 65 E. RANDOLPH SrTItPDffiT\
				

